# Supplementary material for: Annona cherimola Seed Extracts Trigger an Early Apoptosis Response and Selective Anticlonogenic Activity against the Human Gastric Carcinoma Cell Line SNU-1
Source: Molecules. 2023 Oct 2;28(19):6906. doi: 10.3390/molecules28196906 (PMC10574650; doi:10.3390/molecules28196906)
Supplement: Supplementary file 1 [file molecules-28-06906-s001.zip › molecules-2481076-supplementary.pdf]

Supplementary Materials

Annona cherimola Seed Extracts Trigger an Early Apoptosis Response and Selective Anticlonogenic Activity against the Human Gastric Carcinoma Cell Line SNU-1

Johan Macuer-Guzmán [1,2], Claudia Giovagnoli-Vicuña [3], Giuliano Bernal [4], Lorena Lobos-González [5], Erwin de la Fuente-Ortega [6], Michael Araya-Castillo [7] and Cristian Ibáñez [1,8,*]

[1] Laboratorio de Silvigenómica y Biotecnología, Departamento de Biología, Facultad de Ciencias, Universidad de La Serena, Avenida Raúl Bitrán 1305, Casilla 599, La Serena 1700000, Chile; johan.macuer@udalba.cl

[2] Facultad de Ciencias Agropecuarias, Universidad del Alba, Cuatro Esquinas 060, 1700000 La Serena, Chile

[3] Departamento de Química Inorgánica, Facultad de Química y de Farmacia, Pontificia Universidad Católica de Chile, Avenida Vicuña Mackenna 4860, Macul, Santiago 7810000, Chile; ccgiovagnoli@uc.cl

[4] Laboratorio de Biología Molecular y Celular del Cáncer (CáncerLab), Departamento de Ciencias Biomédicas, Facultad de Medicina, Universidad Católica del Norte, Larrondo 1281, Coquimbo 1781421, Chile; gbernal@ucn.cl

[5] Centro de Medicina Regenerativa, Facultad de Medicina-Clínica Alemana, Universidad del Desarrollo, Avenida las Condes 12438, Lo Barnechea, Santiago 7710162, Chile; llobos@udd.cl

[6] Laboratorio de Estrés Celular y Enfermedades Crónicas no Transmisibles, Núcleo de Investigación en Prevención y Tratamiento de Enfermedades Crónicas no Transmisibles (NiPTEC), Departamento de Ciencias Biomédicas, Facultad de Medicina, Universidad Católica del Norte, Coquimbo 1781421, Chile; edelafuente@ucn.cl

[7] Centro de Investigación y Desarrollo Tecnológico de Algas (CIDTA), Facultad de Ciencias del Mar, Universidad Católica del Norte, Larrondo 1281, Coquimbo 1781421, Chile; mmaraya@ucn.cl

[8] Instituto Multidisciplinario de Investigación y Postgrado, Universidad de La Serena, Avenida Raúl Bitrán 1305, Casilla 599, La Serena 1700000, Chile

* Correspondence: cibanez@userena.cl; Tel.: +56-51-2204309 or +56-51-2204383

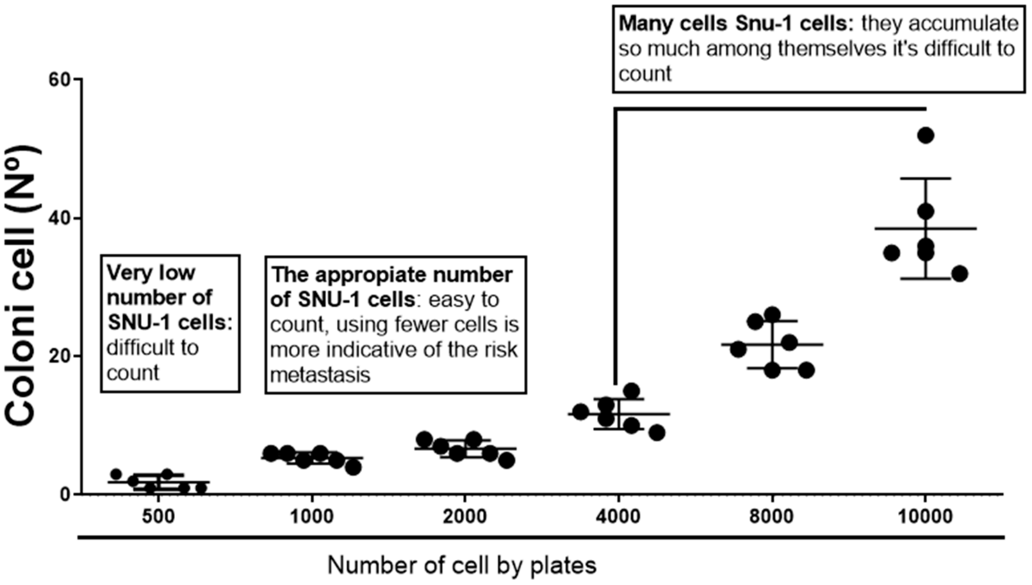

Figure S1. Determination of appropriate number of SNU-1 cells for anchored growth independent assay.

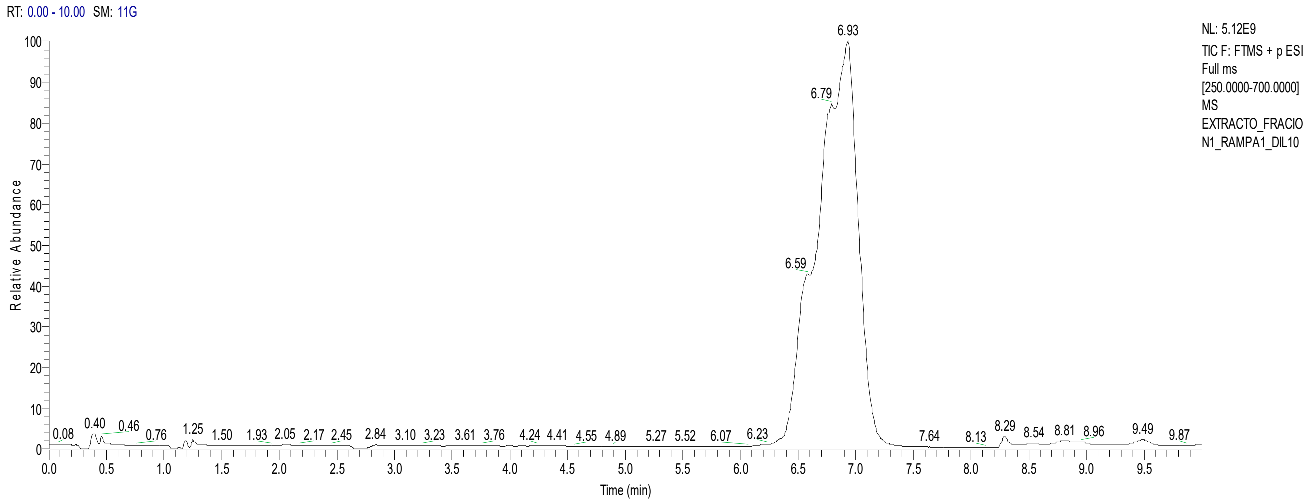

Figure S2. EMCHS extract chromatogram, full scan mode.

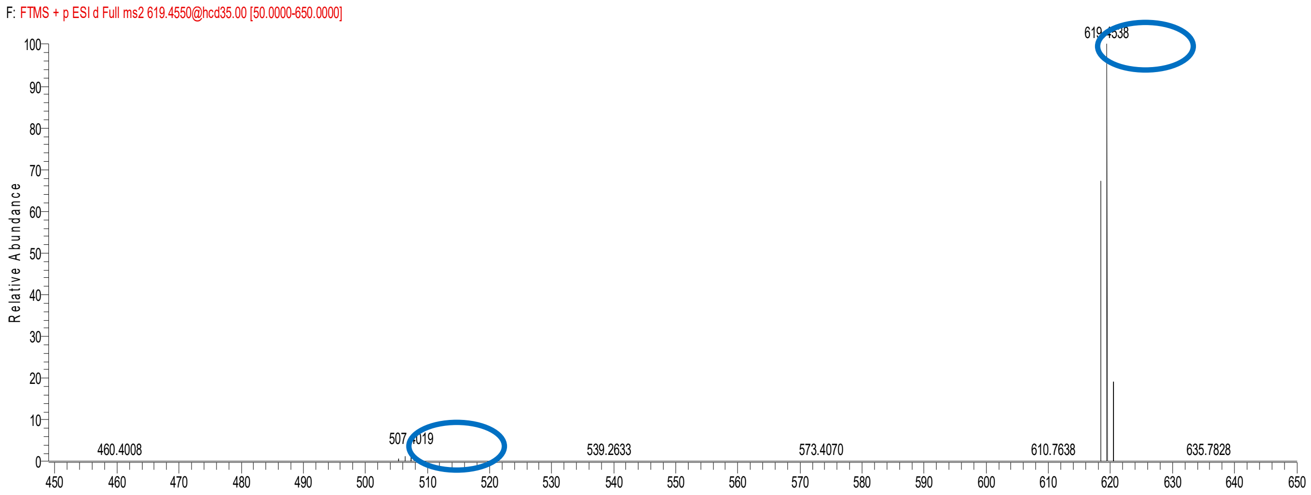

Figure S3. Production of mass spectra (MS/MS) adduct [M+Na]$^+$ of annonacine.

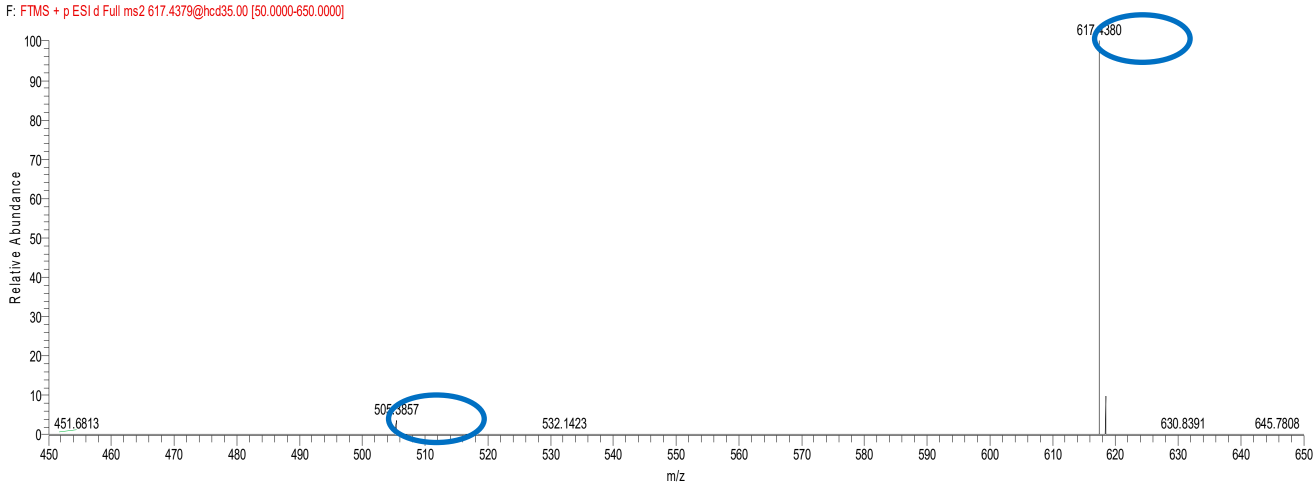

Figure S4. Production of mass spectra (MS/MS) of adducts [M+Na]$^+$ of annonacinone.
